# Technostress at work during the COVID-19 lockdown phase (2020–2021): a systematic review of the literature

**DOI:** 10.3389/fpsyg.2023.1173425

**Published:** 2023-04-25

**Authors:** Maria Emilia Bahamondes-Rosado, Luis Manuel Cerdá-Suárez, Gino Félix Dodero Ortiz de Zevallos, Juan Felipe Espinosa-Cristia

**Affiliations:** ^1^Newman Escuela de Postgrado, Universidad Internacional de La Rioja (UNIR), Logroño, Spain; ^2^Facultad de Empresa y Comunicación, Universidad Internacional de La Rioja (UNIR), Logroño, Spain; ^3^Departamento de Ingeniería Comercial, Universidad Técnica Federico Santa María, Valparaíso, Chile

**Keywords:** psychological stress, information technology, occupational hazards, COVID-19, technostress, home-office, telecommuting, remote work

## Abstract

Technostress is a psychosocial phenomenon associated with the use of technologies to the detriment of health, the same one that during the pandemic was accelerated in the work considering home confinement. This work aims to systematize the main research on the impact of technostress at work during the severe confinement stage of the pandemic, between the years 2020 and 2021, with the purpose of identify and evaluate its main determining factors. A systematic review of the literature was carried out during COVID-19, using the words “technostress work COVID-19.” The works found focus mainly on analyzing the creators and inhibitors of technostress in workers, as well as the main consequences of the materialization of this technological risk associated with work performance during the stage of confinement by COVID-19. Techno invasion and techno overload were identified as the main techno stressors, with the main type of technostress appreciated in the literature being techno fatigue. Technostress is identified as a problem that had direct and relevant effects during the season of severe confinement and remote work at home due to COVID-19; highlighting techno fatigue as the most frequent type of stress, and techno stressors such as techno invasion and overload as the ones that presented the highest incidence.

## 1. Introduction

For Despite providing a functional solution for human interaction, information, and communication technologies (ICT) have also proven to be a potential problem for the health of organizations’ human resources, as more advanced technological innovation and improved functionality go hand-in-hand with greater technological stress. Technostress is defined as the stress that people experience as a result of their use of information and communication systems and technologies (Tarafdar and Gordon, 2007; Tarafdar et al., 2014; Chen, 2015). It is a global phenomenon, which has been demonstrated by academic research and is experienced by workers across national borders and cultures (Chen, 2015).

As is well known, a series of cases of atypical pneumonia were reported in the city of Wuhan (China) in December 2019, which were triggered by a new coronavirus called SARS-CoV-2 that causes COVID-19 disease (Mojica-Crespo and Morales-Crespo, 2020). In March 2020, a pandemic was declared in response to the exponential rise in cases in several countries (Ramírez et al., 2021), with consequences for education, society, the economy, the public authorities and economic and social activity more broadly. The widespread virtualization that emerged during this period gave rise to a range of psychosocial problems affecting workers’ health, including technostress. The term “technostress” was coined by Brod (1984), who defined it as an individual’s inability to adapt to the use of ICT in a healthy manner due to factors such as age, prior experience of technology, connection time, perception, etc., with an impact on their performance.

Remote working entails working outside the company’s physical premises using information technologies (Chong et al., 2020). As noted above, the emergence of the SARS-CoV-2 pandemic forced the world into lockdown and provided ample opportunity for remote working, which allowed work to continue remotely using technology in most administrative roles in both the public and private sector. Despite this, most people were unprepared for remote working or had rarely experienced it prior to the pandemic and were affected in different ways by the sudden virtual, remote nature of their work; one of the outcomes of this shift to remote working was technostress (Tuan, 2022).

The conservation of resources theory to understand stress among employees working remotely during the COVID-19 pandemic began recently (Colquhoun et al., 2014). They drew on the work of Wang and Shu (2008), Tarafdar et al. (2010), and Salazar-Concha et al. (2021), who define resources as elements such as energy and emotions that are perceived by individuals as allowing them to achieve their objectives. In their study, the disruption caused by the COVID-19 pandemic was viewed by Colquhoun et al. (2014) as a period when remote workers experienced a fluctuation in their roles and tasks, and therefore, in their resources.

Three types of technostress have been identified by the literature (La Torre et al., 2020). The first is techno-anxiety, which is perhaps the most well-known type of technostress. It refers to a state in which people experience high levels of physiological arousal, increased blood pressure and unease due to their current or future use of ICT. The second is techno-fatigue, which is characterized by tiredness and cognitive exhaustion because of the use of ICT and can be exacerbated by feelings of wariness and inadequacy in relation to its use. Finally, the third is techno-addiction, which is a phenomenon whereby people feel a constant, obsessive, compulsive need to use ICT everywhere, all the time, when carrying out their activities, evaluating work, providing feedback, and caring for their families.

Meanwhile, a model of technostress that differentiates between stressors and strains was developed by Tarafdar and Gordon (2007). The model identifies a scale of factors, including:

1. Techno-invasion: technology is invasive and plays a central part in workers’ everyday lives.
2. Workers struggle to separate their personal lives from their work as they are always “connected.” This affects their free time and their privacy.
3. Techno-overload: an increase in workload forces employees to maintain an unsustainable pace. Technology brings with it a significant amount of work-related information that individuals feel pressured to address.
4. Techno-complexity: technology can be difficult to use. Workers feel that they are inadequate.
5. Techno-insecurity: workers do not feel secure within their companies. They believe that any distraction from their objectives will lead to their dismissal.
6. Techno-uncertainty: changes and upgrades to ICT oblige workers to constantly learn how to use it.

In the same line of research, Ayyagari et al. (2011) adapted the previously identified technostress creators into five techno-stressors: work overload (i.e., perception that the assigned job exceeds capabilities or skills), role ambiguity (i.e., lack of information to perform the job), job insecurity (i.e., perception of threat of losing one’s job; like techno-insecurity), work–home conflict (i.e., perceived conflict between home and work demands), and invasion of privacy (i.e., perception of a compromised privacy). The literature on technostress has identified several factors that can influence and exacerbate the stressors affecting individuals, as La Torre et al. (2020) notes. This includes individual differences, organizational characteristics and perceptions relating to technological characteristics (Hutton et al., 2015; Lockwood et al., 2017; Abbas et al., 2020; Carillo et al., 2021).

Against this backdrop, this study aims to systematically review recent studies on the impact of different techno stressors at work during the COVID-19 lockdown phase in the early years of the pandemic in order to identify positive and negative impacts during these types of lockdowns. This line of research makes an important contribution to ascertaining the variables of technostress that are relevant to work, with a focus on the period of lockdowns triggered by the public health situation in countries where the topic in question has been studied specifically. Evaluating these variables represents a fruitful avenue for exploring their relevance within organizations.

In the present paper, the general objective is defined as follows: to systematically review the main studies on technostress and its relationship with work activities during the COVID-19 lockdown period in 2020–2021. Specific objectives are:

1. To qualitatively analyze the techno stressors caused by the COVID-19 pandemic during the period.
2. To ascertain the main impacts of technostress on work caused by the COVID-19 pandemic during the study period, in accordance with the systematic review of the literature.
3. To identify the type of technostress that had the greatest impact on work during the period of the COVID-19 pandemic under study, in accordance with the systematic review of the literature.

## 2. Materials and methods

**FIGURE 1:**
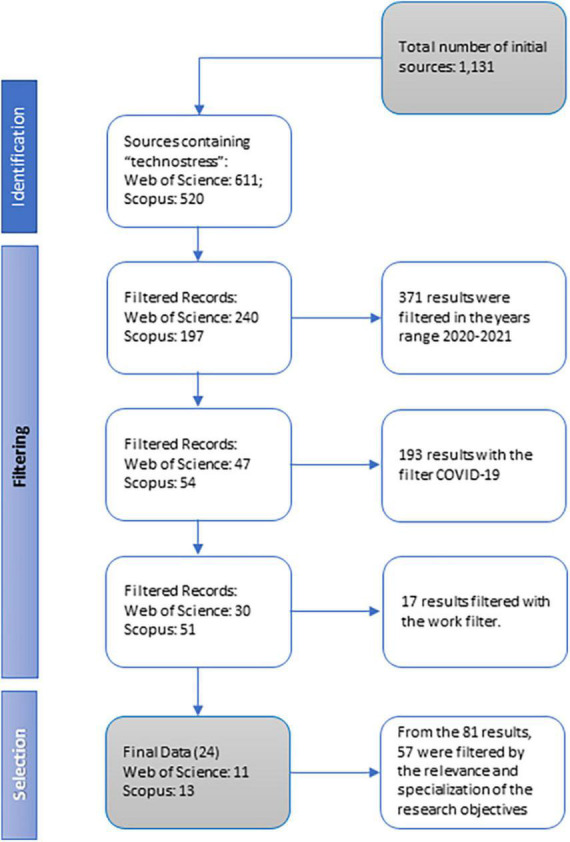
Flow diagram (PRISMA 2020 version). Compiled by the authors, following Oksanen et al. (2021) and the PRISMA 2020 statement: an updated guideline for reporting systematic reviews.

This study employed an exploratory, non-cross-sectional, qualitative, and systematic design. A comparative analysis of the information presented in recent studies associating variables of technostress with work during the COVID-19 lockdowns in 2020–2021 was carried out to systematically review the main results obtained and identify relevant themes. All studies investigating these research topics were searched through two database [SCOPUS and Web of Science (WOS)] and all entries were imported in Zotero to remove duplicates, after which titles and abstracts were screened according to predefined eligibility criteria (the full description of the methodology is reported in Figure 1 and Supplementary Table 1). Due to the high heterogeneity of the studies included, a meta-analytic summary of the findings was not feasible.

From 1,131 studies identified through the initial research, after excluding duplicates, conference papers, and book chapters and reviewing title and abstract screening, 24 articles were included in the present study (PRISMA flowchart is reported in Figure 1). Importantly, although we may use causal-like terms in describing the results, the reader should keep in mind that all the studies (apart from four of them regarding cultural aspects and validation of scales adapted to other countries) made use of a cross-sectional design.

### 2.1. Research questions

The methodology was informed by the following specific research questions:

1. How did technostress affect people’s work during the lockdown phase of the COVID-19 pandemic? (Q1).
2. What were the main techno stressors affecting work during the lockdown period? (Q2).
3. Which studies have provided evidence of these impacts at the global level? (Q3).

To answer these questions, the methodology recommended in the PRISMA 2020 statement was used to systematically tabulate the main results of recent studies published in the field covered by this review. Based on the study variables identified, the bibliographic references were tabulated in accordance with the following criteria: (a) temporal scope: 2020–2021 (associated with the COVID-19 pandemic); (b) type of publication: indexed journals; (c) field: labor psychology; and (d) key topics: technostress and work. These criteria allowed relevant information to be obtained on the effects of technostress on work during the COVID-19 pandemic.

Although many aspects must be taken into consideration in the exceptional transition to extensive virtual work, which included increased workload among other external factors most relevant, in the analytical process of this paper a top-down approach was taken. In other words, the lines of research studied around the start of the COVID-19 pandemic (from early February 2020) were followed. Depending on their continuance, the analysis then considered the second wave of the pandemic (December 2020–February 2021), identifying different remote working practices according to the type of organization, industry, and public or private sector. This was then linked to prior knowledge of technostress as a factor associated with excessive use of technology and of the reorganization of work settings and activities. This process gave rise to inferences that were shared between the authors, allowing several prior assumptions to be established to determine and reveal the development of the phenomenon of technostress and remote work during the study period. The study did not focus on a specific sector; instead, it explored the effects of technostress in remote working situations that were imposed during the lockdown phase.

Once the study area had been defined, electronic records were identified through a systematic bibliographic search carried out by inserting the keyword extraction algorithm into the scientific databases used in the study, SCOPUS and WOS, which were selected for their reliability and relevance to the social sciences. The study was conducted in accordance with the guidelines for systematic reviews published in the 2020 edition of the PRISMA statement (Preferred Reporting Items for Systematic Reviews and Meta-Analyses).

The research took place between December 2021 and February 2022 and included qualitative studies containing keywords such as “technostress,” “work,” “COVID-19,” “2020,” and “2021.” These keywords and their scope were selected to fulfill the study objectives: to ascertain the impact of technostress at work during the COVID-19 pandemic and its relevance as a framework to guide action by organizations.

### 2.2. Inclusion criteria

The inclusion criteria used to select the articles were: written in English or Spanish; keywords; title and summary in English; translated with Google Translate. These criteria were chosen to link the study objective to the research areas, with a series of exclusion criteria applied to the latter. The exclusion criteria used to select the articles were: taking an educational perspective (e.g., technostress among students); taking a family perspective (e.g., technostress among children, elderly people, etc.); taking a clinical perspective (e.g., associated pathologies); review articles; gray literature; letters to the editor; conference summaries, and lectures. No geographical restrictions were applied as studies from different continents relating to the variables and research areas (technostress at work during the 2020–2021 period of the COVID-19 pandemic) found in the SCOPUS and WOS databases were taken into consideration in the review.

Table 1 shows the checklist drawn up in accordance with the PRISMA 2020 guidelines, which contains the main factors considered in the qualitative systematic review of the selected studies. Figure 1 shows the development of a filter based on the research criteria applied in this study.

**TABLE 1:** Checklist – PRISMA 2020 guidelines.

Item (PRISMA 2020)		Description
**Title**		
Title	1	Technostress at work during the COVID-19 lockdown phase (2020–2021): a systematic review of the literature
Introduction		
Objective	2	To conduct a systematic review of the main studies on technostress and its relationship with work during the 2020–2021 period of the COVID-19 pandemic.
**Methods**		
Eligibility criteria	3	Technostress; work; COVID-19; 2020–2021 To access the selected studies, a search was carried out in the databases of articles and indexed studies, starting with a number, then applying the filters in the electronic system. The first filter was “technostress,” then “2020–2021,” then “COVID-19” and finally, “work” (PICOS was considered)
Information sources	4	SCOPUS WOS
Risk of bias in studies	5	The risk of bias in the selected studies was largely associated with the variety of business sectors and markets explored in them, including the education sector (on the work of teachers), the corporate sector (on Support or Operations departments), the public sector (on organizations, agencies, and locations), etc. This diversity could affect the uniformity of the results due to bias from studies seeking to associate the phenomenon with a specific sector. Finally, bias may also arise from the different countries covered by the studies as they may have different cultures when it comes to adapting to technology and this may impact on perceptions of technostress.
Study risk of bias assessment	6	In order to evaluate the quality of the sample obtained in the systematic qualitative review, the JBI-Qualitative Critical Appraisal Checklist was applied (see Table 2). The evaluation considered the characteristics of the articles that complied with the JBI method, which assesses aspects such as congruity between study methodologies, qualitative methods, interpretation of the results, adequate representation of participants and ethical approval, among others.
**Outcomes**		
Study selection	7	Supplementary Table 1 shows the studies that explore technostress as a main theme in work-related situations in any sector or industry during the COVID-19 pandemic between 2020 and 2021. The origin of the studies was highly diverse, with samples from China, Korea, India, Finland, Italy, USA, Germany, Spain, Colombia, and Mexico.
Results of syntheses	8	Among the most important findings of the qualitative systematic review, workers were found to experience greater techno-fatigue as they lacked control over their working hours and were unaccustomed to working remotely: the results concerning teachers, lecturers, and professors, who had to adapt their teaching methodologies in order to work remotely, are particularly striking. Techno-overload was also observed among workers, who had to adapt suddenly and unexpectedly to new ways of working at home during the COVID-19 lockdown. In both cases, the results displayed a series of negative effects on occupational health, including reduced productivity levels.
**Discussion**		
Limitations of evidence	9	The evidence analyzing the impact of technostress at work during the COVID-19 pandemic was obtained using different methodological approaches. It also relates to a diverse range of specialist sectors. In order to evaluate the quality of the sample obtained in the systematic qualitative review, the JBI-Qualitative Critical Appraisal Checklist was applied (see Table 2).
Interpretation	10	See section “4.3. Workers and remote working” of this study.
**Other**		
Support	11	
The register	12	

Compiled by the authors with reference to the PRISMA 2020 checklist for systematic reviews. This checklist uses the same items as those included in the PRISMA statement for systematic reviews published in 2013 by Colquhoun et al. (2014), but it has been revised to ensure that the terms used are consistent with the PRISMA 2020 statement.

**TABLE 2:** Table of articles analyzed using the JBI-Qualitative Critical Appraisal Checklist.

Results of the critical analysis of the articles included in the study using the JBI-Qualitative Critical Appraisal Checklist											
**References**	**Q1**	**Q2**	**Q3**	**Q4**	**Q5**	**Q6**	**Q7**	**Q8**	**Q9**	**Q10**	**T**
Estrada-Muñoz et al., 2021	U	Y	Y	Y	Y	Y	Y	Y	Y	Y	9
Panisoara et al., 2020	Y	Y	Y	Y	Y	Y	Y	Y	Y	Y	10
Chou and Chou, 2021	U	Y	Y	Y	Y	Y	Y	Y	Y	Y	9
Becker et al., 2022	Y	Y	Y	Y	Y	Y	Y	Y	Y	Y	10
Kaltenegger et al., 2020	Y	Y	Y	Y	Y	Y	Y	Y	Y	Y	10
Sinha et al., 2021	Y	Y	Y	Y	Y	Y	Y	Y	Y	Y	10
Zito et al., 2021	Y	Y	Y	Y	Y	Y	Y	Y	Y	Y	10
Gabr et al., 2021	Y	Y	Y	Y	Y	Y	Y	Y	Y	Y	10
Hwang et al., 2022	Y	Y	Y	Y	Y	Y	Y	Y	Y	Y	10
Spagnoli et al., 2020	Y	Y	Y	Y	Y	Y	Y	Y	Y	Y	10
Taser et al., 2022	Y	Y	Y	U	Y	Y	Y	Y	Y	Y	9
Oksanen et al., 2021	Y	Y	Y	Y	Y	Y	Y	Y	Y	Y	10
González-López et al., 2021	Y	Y	Y	Y	Y	Y	Y	Y	Y	Y	10
Estrada Araos and Gallegos Ramos, 2022	Y	Y	Y	Y	Y	Y	Y	Y	Y	Y	10
Kim and Kang, 2020	Y	Y	Y	Y	Y	Y	Y	Y	Y	Y	10
Brooks et al., 2020	Y	Y	Y	Y	Y	Y	Y	Y	Y	Y	10
Carillo et al., 2021; Chung et al., 2023	Y	Y	Y	Y	Y	Y	Y	Y	Y	Y	10
Christ-Brendemühl and Schaarschmidt, 2020	Y	Y	Y	Y	Y	Y	Y	Y	Y	Y	10
Califf and Brooks, 2020	Y	Y	Y	Y	Y	Y	Y	Y	Y	Y	10
Califf et al., 2020	Y	Y	Y	Y	Y	Y	Y	Y	Y	Y	10

Questions: T, total value; Y, yes; N, no; U, unclear; N/A, not applicable. Compiled by the authors following the JBI model adapted from La Torre et al. (2020).

**TABLE 3:** Systematic review of articles with answers to our specific research questions.

Group	How did technostress affect people’s work during the lockdown phase of the COVID-19 pandemic? (Q1)	What were the main techno stressors affecting work during the lockdown period? (Q2)	Studies providing evidence of these impacts at the global level (Q3)
1	Social media communication (SMC) at work increased and predicted higher technostress. Technostress and work exhaustion decreased among workers already accustomed to using social media communication (SMC) at work before the crisis. The study focused on the ways in which senior citizens made use of social networking sites and various digital platforms to manage their lives better, analyzed the technology adoption process, and examined the extent of the impact of technology on elderly people’s lives and the permanent nature of this change. The analysis points to an increase in digital life among elderly people. The technology adoption process progressed in stages, from complete confusion to relative ease, significantly reducing elderly people’s loneliness and bringing about a relatively stable change to their way of life. A competitive mediation effect was found, where the direct effect of demands on productivity is in the opposite direction to the indirect effect. Both active-functional and dysfunctional coping reduce the extent to which demands lead to strain.	Social media communication (SMC) at work, and work exhaustion. Use of technology via platforms of social networks, and ICTs for communication. Strain linked to ICT use and lack of behavior-based functional and dysfunctional coping strategies.	(Oksanen et al., 2021): COVID-19 crisis and digital stressors at work: a longitudinal study on the Finnish working population. (Sinha et al., 2021): Technology: saving and enriching life during COVID-19. (Becker et al., 2022): Mitigating the negative consequences of ICT use: the moderating effect of active-functional and dysfunctional coping.
2	The lockdown imposed due to the COVID-19 pandemic prompted a rethink of the teaching-learning process, with teachers responding to the situation without prior planning using their own resources. Positive reciprocal family-work interactions and their relationship with organizational support were observed, with differences by gender: women displayed more negative perceptions of the impact on the family. Teachers at private and subsidized schools expressed higher levels of perceived support than those at public schools. Technological pedagogical knowledge, self-efficacy, intrinsic and extrinsic work motivation, and occupational stress (i.e. burnout and technostress) are key dimensions to explain intentions among in-service teachers to continue to use online-only teaching. Intrinsic work motivation has the most direct significant effect on teachers’ online-only teaching, followed by technological pedagogical knowledge, self-efficacy, and occupational stress as significant but less relevant predictors. Intrinsic work motivation was positively associated with technological pedagogical knowledge, self-efficacy, and negatively associated with extrinsic work motivation. High levels of authoritarian leadership had an exacerbating effect and low levels of authoritarian leadership had a protective effect on the relationship between workaholism and technostress in the group of fully remote workers. Therefore, authoritarian leadership should be avoided, and leaders should be trained to raise awareness of its impact. Privacy concerns aggravate teachers’ technostress in online teaching. Self-efficacy in online pedagogy would alleviate teachers’ technostress. School support matters less in teachers’ intentions to continue online teaching. Technostress, self-efficacy, and school support are related to teachers’ intentions to continue teaching online to different extents at different teaching levels. The teachers’ preference for online teaching lies in adequate teaching resources and flexibility. Technostress is positively associated with psychophysical disorders, whereas self-efficacy is negatively associated. As regards the mediated effects, the results showed negative associations between organizational communication and psychophysical disorders through both technostress and self-efficacy. Potential protective role of organizational communication, which could mitigate the effect of technostress and enhance self-efficacy, a personal resource that helps reduce psychophysical disorders. Organizational communication is positively associated with self-efficacy and negatively associated with technostress and psychophysical disorders. Relationship between technostress and employee performance, taking training and creative self-efficacy as boundary conditions. Technostress had a positive effect on employees’ performance and both training and creative self-efficacy significantly moderated the relationship.	Family-work interactions and lack of organizational support. Lack of technological pedagogical knowledge, lack of self-efficacy, lack of intrinsic and extrinsic work motivation, and occupational stress. Authoritarian leadership and workaholism. Privacy concerns and lack of self-efficacy. Lack of organizational communication, lack of self-efficacy, and psychological disorders. Inflexibility in the form of remote working, lack of training and creative self-efficacy.	(Brooks et al., 2020): Technology addictions and technostress: An examination of the US and China. (Kaltenegger et al., 2020). Association of working conditions including digital technology use and systemic inflammation among employees: Study protocol for a systematic review. (Panisoara et al., 2020): Motivation and continuance intention towards online instruction among teachers during the COVID-19 pandemic: the mediating effect of burnout and technostress. (Spagnoli et al., 2020): Workaholism and technostress during the COVID-19 emergency: the crucial role of leaders on remote working. (Chou and Chou, 2021): A multigroup analysis of factors underlying teachers’ technostress and their continuance intention toward online teaching. (Zito et al., 2021): Does the end justify the means? The role of organizational communication among work-from-home employees during the COVID-19 pandemic. (Hwang et al., 2022): Impact of regulatory focus on security technostress and organizational outcomes: The moderating effect of security technostress inhibitors.
3	Teachers displayed techno-anxiety and techno-fatigue. Combining both manifestations, 6.8% of teachers are found to be experiencing technostress. Finally, fatigue and anxiety factors are higher among female teachers. The analysis, based on an exploration of a system of archetypes of social media use, presents insights into contemporary technostress management as a new approach offering opportunities to optimize prevention plans. The results provide valid, reliable measures indicating the major impact of technostress on students’ individual spheres and show a significant relationship between user type and techno-anxiety.	Techno-anxiety and techno-fatigue. Techno-anxiety and archetypes of social media use, moderated by type of individual, social, and professional spheres.	(Estrada-Muñoz et al., 2021): Technostress of Chilean teachers in the context of the COVID-19 pandemic and teleworking. (González-López et al., 2021): Overwhelmed by technostress? Sensitive archetypes and effects in times of forced digitalization.
4	Teachers experience moderate levels of technostress, very high levels of work performance and job satisfaction, and high levels of career commitment. Technostress differed by age, gender, marital status, and teaching experience. Technostress had a significant negative relationship with work performance. The results showed that techno-complexity, techno-invasion, and techno-overload are the main factors affecting negative psychological responses during the COVID-19 pandemic. Among them, techno-overload was found to have the most significant influence. This is due to the lack of instant feedback on workload allocations and the lack of an adjustment period with the sudden shift to teleworking. In the case of techno-complexity, employees appear to experience difficulties acquiring new technical skills. Finally, techno-invasion also proved significant as home-related activities infiltrated the working environment. One type of smart work, teleworking, was suggested as a preventive measure and the technology urgently introduced due to the COVID-19 pandemic is expected to increase the prevalence of technostress. Smart work introduced to improve performance was causing technostress and that technostress had a negative impact on productivity at work. Technostress has a negative influence on productivity. The results point to a significant influence of crisis-specific variables such as professional isolation, telework environment, increased workload, and stress. Predictors of work overload in the multivariate regression were female gender and a work environment with poor Wi-Fi. Being female, working at a theoretical college, being a lecturer or higher and having poor Wi-Fi were predictors for invasion. High levels of technostress were significantly influenced by age, higher ranking roles, female gender and a poor work environment. Participants who were female, living in rural areas, occupying a lecturer role or higher position, had poor Wi-Fi in their work environment and lacked technical training obtained significantly higher scores on the technostress. The results confirmed positive relationships between workload, techno stressors, work-family conflict and behavioral stress. The role of remote working conditions was also analyzed. Technostress and loneliness serially mediated the relationship between remote e-working and flow, which result in lower levels of flow at work.	Work-home conflict, work overload, and invasion of privacity. Techno-complexity, techno-invasion, and techno-overload. Smart work (teleworking) and technology. Work overload and work-home conflict. Professional isolation, telework environment, and workload. Work overload and work environment with poor Wi-Fi influenced by age, position roles, and gender. Teleworking, workload, work-family conflict, and behavioral stress. Technostress and loneliness.	(Califf and Brooks, 2020): An empirical study of techno-stressors, literacy facilitation, burnout, and turnover intention as experienced by K-12 teachers. (Califf et al., 2020): The bright and dark sides of technostress: A mixed-methods study involving healthcare IT. (Christ-Brendemühl and Schaarschmidt, 2020). The impact of service employees’ technostress on customer satisfaction and delight: A dyadic analysis. (Kim and Kang, 2020): The impact of technostress on counter-productivity. (Carillo et al., 2021): Adjusting to epidemic-induced telework: Empirical insights from teleworkers in France. (Gabr et al., 2021): Effects of remote virtual work environment during COVID-19 pandemic on technostress among Menoufia University Staff, Egypt: A cross-sectional study. (Estrada Araos and Gallegos Ramos, 2022): Tecnoestrés en el contexto educativo: Un problema emergente durante la pandemia COVID-19. (Taser et al., 2022): An examination of remote e-working and flow experience: The role of technostress and loneliness.

### 2.3. Evaluation and methodological quality

Following Torres (2021), the selected articles were evaluated independently by two reviewers. Any differences between the two reviewers were resolved through discussion or, if a consensus could not be reached, by involving a third reviewer. For the methodological review, the articles were evaluated using the JBI Critical Appraisal Checklist for Qualitative Research. More specifically, this instrument assesses methodological quality via a thorough analysis of the following elements in question format (Q): philosophical perspective adequately established (Q1); congruity between the research methodology and the research question (Q2); qualitative methods (Q3); data analysis (Q4) and interpretation of results (Q5); adequate reference to researcher’s cultural or theoretical paradigm (Q6); researcher’s influence on research addressed (Q7); adequate representation of participants and their voices (Q8); ethical approval by an appropriate body (Q9); and adequate presentation and interpretation of final conclusions (Q10). All articles with a positive score in 80% of the aforementioned items were included in the study.

## 3. Analysis and results

Supplementary Table 1 on the techno stressors associated with work around the world that were identified in this study are presented below.

One important aspect of the results analysis in this systematic literature review is related with the thematic categorization and meta-aggregation of the data following the PRISMA methodology. In this study, the findings were summarized, organized, and aggregated into themes of relevance and interest. Four main thematic categories were identified, allowing the main objective of this systematic literature review to be fulfilled: Thematic category 1: “relevance of technostress at work”; Thematic category 2: “impact of COVID-19 on remote work”; Thematic category 3: “workers and remote working”; and Thematic category 4: “contextual barriers to remote working.” In the following section, the main results obtained from the analysis are described and discussed.

## 4. Discussion

### 4.1. Relevance of technostress at work

Regarding remote work, which represents the focus of this study, high levels of technostress were significantly influenced by age, higher ranking roles, female gender, and a poor working environment (Benlian, 2020; Cheikh-Ammar, 2020). The analysis clearly points to an increase in digital life among elderly people. Higher levels of perceived inadequacy and skepticism were observed among teachers aged 46 and over, for example (Dias Pocinho and Costa Garcia, 2008; Kaltenegger et al., 2020). Indeed, technology adoption process progressed in stages, from complete confusion to relative ease, significantly reducing elderly people’s loneliness and bringing about a relatively stable change to their way of life (Domínguez-Torres et al., 2021; Sinha et al., 2021).

One of the most frequent samples found, during the systematic review, was teachers working in the education sector. Some of those studies explored the impact of technostress on their work. Teachers experienced moderate levels of technostress and very high levels of professional performance and satisfaction, while technostress was found to have a significant negative association with professional performance (Pandey and Pal, 2020; González-López et al., 2021; Oksanen et al., 2021). One of the studies showed that 11% of teachers display techno-anxiety and 7.2% techno-fatigue (Hinojosa López et al., 2021). Privacy concerns aggravate teachers’ technostress in online teaching. Self-efficacy in online pedagogy has been shown to alleviate teachers’ technostress (Khedhaouria and Cucchi, 2019; Beare et al., 2020; Kalischko et al., 2020; Sinha et al., 2021; Hwang et al., 2022).

Technostress was found to be a complex phenomenon with an impact on users of information systems and its complexity and impact have been neglected in the systematic study carried out in this work. Technostress is a crucial area of research, which must seek to establish the true psychosocial impact on workers (techno-uncertainty, techno-complexity, and techno-addiction, which are direct causes of technostress), who experience strains that may affect their health in the form of burnout, anger and anxiety, for example, reducing their professional satisfaction and productivity. In some individual cases, workers may display technostress inhibitors – technological self-sufficiency (La Torre et al., 2019; Ju and Sook, 2020; Zito et al., 2021).

### 4.2. Impact of COVID-19 on remote work

The COVID-19 virus has had an unprecedented impact on people, organizations, and industries (Jung and Kim, 2020; Kaltenegger et al., 2020) affecting individuals’ social, professional, and financial lives, imposing changes to the social order, and reducing wellbeing and quality of life among employees (Martínez, 2020). Workplace closures and social distancing measures in companies intended to stop the spread of the virus, such as remote working and digitalization, have presented new challenges for workers. Research has shown that digital remote working during the COVID-19 pandemic has led to technostress among workers (Brooks et al., 2020; Nisafani et al., 2020; Korzynski et al., 2021) which is defined as the stress experienced by individuals due to their inability to cope with the demands of using information technology (Tarafdar and Gordon, 2007; Nimrod, 2022).

The results point to a significant influence of crisis-specific variables such as professional isolation, remote work environment, increased workload, and stress. They also show how detachment mediates the effect of telepressure on wellbeing and how technostress inhibitors moderate the effect of technostress creators on wellbeing (La Torre et al., 2019; Becker et al., 2022; Nasirpouri Shadbad and Biros, 2022). Analysis of the moderating effect shows that control behavior has a negative effect and technostress a positive effect on alleviating technostress (Kim and Kang, 2020; Nyland et al., 2020; Panisoara et al., 2020).

Regarding techno stressors, techno-complexity, techno-invasion, and techno-overload were found to be the main factors producing negative psychological responses during the COVID-19 pandemic, with techno-overload having the most significant influence. This is due to the lack of instant feedback on workload allocations in remote working and the absence of an adjustment period. In the case of techno-complexity, employees appear to experience difficulties in acquiring new technical skills. Finally, techno-invasion also proved significant as home-related activities infiltrated the working environment (Turel and Gaudioso, 2018; Califf et al., 2020; Pullins et al., 2020; Ploder et al., 2021; Soror et al., 2022).

### 4.3. Workers and remote working

Respecting to the impact of technostress on organizations, the following results were obtained from the analysis. There was a negative association between organizational communication and psychophysical disorders through both technostress and self-efficacy (Wang and Shu, 2008; Vaziri et al., 2020; Zhao et al., 2020; Tiwari, 2021; Zito et al., 2021). Meanwhile, there was a negative relationship between promotion-focused employees and security technostress creators, with a negative impact on strains such as organizational commitment and compliance intentions. High levels of authoritarian leadership had an exacerbating effect and low levels of authoritarian leadership had a protective effect on the relationship between workaholism and technostress (Ferziani et al., 2018; Brooks et al., 2020; Hwang and Hu, 2020; Luqman et al., 2020; Toscano and Zappalà, 2020; Korzynski et al., 2021). Finally, smart work introduced to improve performance was shown to cause technostress, which had a negative impact on productivity at work (Kaltenegger et al., 2020; Eidman and Basualdo Felleau, 2021).

The results indicate a disparity in workers’ resilience during remote work and highlight the need for support at the organizational level (Vaziri et al., 2020; Pflügner et al., 2021; Taser et al., 2022). Technostress is positively associated with psychophysical disorders, whereas self-efficacy is negatively associated (Wang and Shu, 2008; Zhao et al., 2020; Estrada-Muñoz et al., 2021). Mindfulness led to lower perceived levels of technostress, but it did little to mitigate the effect of perceived techno stressors on burnout. Techno-overload, intensity of daily workload, techno-invasion, and the socioemotional consequences of working beyond normal working hours were found to be technostress creators, while technostress inhibitors did not appear to perform a protective role. Training and creative self-efficacy were useful to control technostress and maintain the performance of lecturers during COVID-19 (Luqman et al., 2020; Estrada-Muñoz et al., 2021).

When transitioning to digital platforms as a social distancing measure during the pandemic, organizations must take technostress among employees into consideration and promote proactive coping strategies. Organizations must offer training in mindfulness and interpersonal care among employees, encouraging them to pay attention to their needs for growth and learning and to seek a balance between their need for growth (promotion-focused) and need for security -prevention-focused (Toscano and Zappalà, 2020).

### 4.4. Contextual barriers to remote working

When it comes to contextual barriers to remote working, limitations were observed in terms of the diverse results and methods used to obtain them. This was influenced by the different countries and their cultures of technology use, as well as by the sectors or areas in which the organizations operated.

Priority was given to studies focusing on the impact of techno stressors on work during the pandemic: these included techno-fatigue caused by excessive technology use during the lockdown period and techno-overload caused by people’s dependency on technology as a key channel for communication.

In terms of contextual barriers, to create a framework for the study that will prove useful in practice, the phenomenon of technostress in relation to work and the use of technology during lockdown must be adequately contextualized to allow subsequent studies to explore techno stressors in depth using specific clinical models/methods.

Although contextual barriers to remote working were significant in the period preceding the sudden onset of the pandemic, the results of this study show that future research on specific psychological factors to determine types of techno stressors will be necessary (Ferziani et al., 2018). Many of the studies included in the review focused on teachers in the education sector, so future research should explore other professional sectors, especially those where remote working has been particularly prominent (Ferziani et al., 2018; Spagnoli et al., 2020).

### 4.5. Thematic categorizations and main findings

This study posed the following research questions: (1) How did technostress affect people’s work during the lockdown phase of the COVID-19 pandemic? (Q1); (2) What were the main techno stressors affecting work during the lockdown period? (Q2); and (3) Which studies have provided evidence of these impacts at the global level? (Q3). According to a GRADE approach to address the answers to these research questions (Schünemann et al., 2019), the literature review was referred previously to a research strategy for assessing certainty of the evidence in terms of the thematic categories: “relevance of technostress at work,” “impact of COVID-19 on remote work,” “workers and remote working,” and “contextual barriers to remote working.” In the following subsection, the main results obtained from the categorization are discussed (answer to Q3 is included in Table 3).

Based on these studies, the present systematic review revealed important results leading to theoretical considerations and evidence on the technostress during the 2020–2021 period. Table 3 regarding systematic review of articles describes answers to our specific research questions. Thus, in response to the first research question (Q1), a range of factors are antecedents of technostress affecting people’s work during the lockdown phase of the COVID-19 pandemic, such as social media communication (SMC) among workers already accustomed to using SMC at work before the crisis, the ways in which people made use of social networking sites and various digital platforms to manage their lives better. Also, relationships between technostress and employee performance are evidenced, taking training and creative self-efficacy as boundary conditions, privacy concerns, and psychophysical disorders. The potential protective role of organizational communication could mitigate the effect of technostress and enhance self-efficacy, a personal resource that helps reduce psychophysical disorders. Additionally, high levels of authoritarian leadership had an exacerbating effect on the relationship between workaholism and technostress. Therefore, authoritarian leadership should be avoided, and leaders should be trained to raise awareness of its impact.

A diverse range of findings are related to techno-anxiety and techno-fatigue. Combining both manifestations, studies present insights into contemporary technostress management as a new approach offering opportunities to optimize prevention plans. Finally, technostress and loneliness serially mediated the relationship between remote e-working and flow, which result in lower levels of flow at work and evidencing a significant influence of crisis-specific variables such as professional isolation, telework environment, and increased workload. The results confirmed positive relationships between workload, techno stressors, work-family conflict, and behavioral stress.

Regarding the second research question (Q2), among the studied constructs, contextual barriers to remote working represent the most consistent portion (eight articles linked to job characteristics: group 4 in Table 3). That is, work-home conflict, and invasion of privacity, technostress and loneliness, professional isolation, telework environment, techno-complexity, techno-invasion, techno-overload, smart work (teleworking), and technology, are symptoms that did not only positively correlate with each other, but they also independently predicted technostress as a broad range of stressors. The same picture emerged concerning organizational environment problems linked to the impact of COVID-19 on remote work, which were generally well investigated (seven articles in total: group 2 in Table 3). In this sense, a range of diverse stressors during the 2020–2021 period are related to the presence of aspects such as inflexibility in the form of remote working, lack of training and creative self-efficacy, privacy concerns, lack of self-efficacy, lack of organizational communication, psychological disorders, lack of technological pedagogical knowledge, lack of self-efficacy, lack of intrinsic and extrinsic work motivation, occupational stress, family-work interactions, lack of organizational support, authoritarian leadership, and workaholism.

Similarly, three articles referred to technology features and strain linked to technology describe the relevance of technostress at work, with stressors such as SMC at work, work exhaustion, use of technology via platforms of social networks, and ICTs for communication, strain linked to ICT use, and lack of behavior-based functional and dysfunctional coping strategies to face these circumstances (group 1 in Table 3). Finally, two studies are related to personal characteristics (group 3 in Table 3), describing stressors such as techno-anxiety, techno-fatigue, techno-anxiety, and archetypes of social media use, moderated by type of individual, social, and professional spheres. Certainly, all last stressors are linked to workers and their presence in remote working.

To sum up, based on our findings it is feasible to sustain technostress affect people’s work during the lockdown phase of the COVID-19 pandemic. Moreover, it is likely that strain linked to technology, occupational and organizational conditions, lead to higher levels of technostress, which, in turn, triggers or increases negative health-related outcomes, and vice-versa (see Table 3).

In terms of a prospective contribution made by this study, intensive, unexpected use of technology had an impact on many people’s performance at work during the COVID-19 lockdown around the world. This experience indicates that the psychosocial factors associated with effective technology use should be addressed directly (by occupational health departments) to implement sustainable mental and physical health policies in the long-term.

## 5. Conclusion

Among the most important findings of the qualitative systematic review conducted for this study, workers were found to experience greater techno-fatigue as they lacked control over their working hours and were unaccustomed to working remotely: the results concerning teachers, lecturers and professors, who had to adapt their teaching methodologies in order to work remotely, were particularly striking. Techno-overload was also observed among workers, who had to adapt suddenly and unexpectedly to new ways of working at home during the COVID-19 lockdown. In both cases, the results displayed a series of negative effects on occupational health, including reduced productivity levels.

High levels of technostress were significantly influenced by age, technical versus professional occupations, female gender, levels of decision-making responsibility and a poor working environment. The analysis clearly points to an increase in digital life among older people, which will require specific analysis in future studies to determine appropriate mitigation measures to address the psychosocial impacts of technostress at work.

Although technostress is not exclusively associated with remote working, it is a disorder that must be adequately detected and treated as it can have a harmful impact on people’s health, giving rise to a range of negative consequences that can trigger related pathologies. Technostress is particularly linked to remote working as it involves measuring the impact of psychosocial effects in a remote working relationship, where concepts associated with the culture of performance-based work are prevalent.

While it is evident that remote working can give rise to greater demands for ICT use and reduced face-to-face interaction, less is known about how this exposure can influence workers’ subjective mental experiences in terms of their concentration and satisfaction at work. However, several studies have provided an insight into the phenomenon associated with techno stressors during the COVID-19 lockdowns and this paper has summarized the main findings relating to this period.

We want to mention some preliminary practical implications at the personal and organizational levels. At the personal level, although are in hot demands nowadays, it is not enough to produce mindfulness programs at work. Such programs do not mitigate the effect of techno stressors on burnout, as we explained before in the discussion section. The proper development of organizations requires a decisive role in the development of stress management at the level of each of its employees. Managers need to be planning, controlling, and organizing continuously the techno-overload, work intensity and put limits for any techno-invasion. Such are relevant decision and managerial variables to avoid any socioemotional consequences of techno stress at working, and overall, to embrace the protective management role in times of deep techno transformations.

At the organizational level and looking at the evidence of the systematic review of the literature, it is possible that a knowledge-diverse work environment becomes an organizational context that could help employees to handle difficult and complex tasks presented by various technologies and alleviate experienced technostress. As the research shows, context is crucial to produce an adequate reduction of technostress, considering the general and cultural organizational aspects related. In this sense, management need to be close involved to monitor and control those interactional and hygienic issues related with the emergency of a culture that nurture the resilience of diversity that will offer flexibility to reduce techno stressors.

This systematic review evaluates studies conducted in different countries on the contingency measures adopted during the COVID-19 pandemic and presents the most relevant findings from these studies. Drawing on the different experiences described in different countries about the topic in question, this study makes a valuable contribution by summarizing the research conducted on manifestations of technostress during the initial lockdown period of the COVID-19 public health crisis from 2020 to 2021. It is hoped that this study will provide a foundation for future research, which will be of interest to different groups involved in effectively managing technostress in organizations, including workers, managers, public authorities, and healthcare professionals.

## Author contributions

MB-R designed the study, wrote the manuscript, and analyzed the data. GD assisted by collecting data. LC-S designed the study, analyzed the data, wrote the text, and reviewed the entire manuscript. JE-C reviewed the data and the procedure, wrote important parts of the manuscript’s discussion and conclusion, and edited the entire manuscript. All authors listed had made a substantial, direct, and intellectual contribution to this research, read, and agreed to the published version of the manuscript.
