# Supplementary material for: Technostress at work during the COVID-19 lockdown phase (2020–2021): a systematic review of the literature
Source: Front Psychol. 2023 Apr 25;14:1173425. doi: 10.3389/fpsyg.2023.1173425 (PMC10167024; doi:10.3389/fpsyg.2023.1173425)
Supplement: Supplementary file 1 [file Table_1.docx]

**Supplementary Table 1**. Systematic review of articles.

|  | **AUTHOR** | **YEAR** | **COUNTRY** | **TITLE** | **DATA BASE** | **SUBJECT OF STUDY** | **METHOD** | **RELEVANT FINDINGS** |
| --- | --- | --- | --- | --- | --- | --- | --- | --- |
| 1 | (Taser et al., 2022) | 2022 | TURKEY | An examination of remote e-working and flow experience: the role of technostress and loneliness | SCOPUS | The aim of this study is to gain insight and to explore the relationship between remote e-working and employee flow experiences by introducing two key stressors: technostress and loneliness. | Data were collected from a survey of 202 employees in the financial services sector in Turkey. | The results revealed that remote e-working experience had a significant and positive effect on flow levels among employees. Technostress and loneliness serially mediated the relationship between remote e-working and flow. The findings contribute to remote e-working research by exploring the consequences of such experiences and introducing two important key stressors, which result in lower levels of flow at work. Practical implications for improving remote e-working conditions and employee wellbeing are provided. |
| 2 | (Oksanen et al., 2021) | 2021 | FINLAND | COVID-19 crisis and digital stressors at work: a longitudinal study on the Finnish working population | SCOPUS | In this longitudinal study, the potential stress effects of social media communication (SMC) at work are investigated. Based on an integrative theoretical model, SMC at work was predicted to burden some workers, while those who were accustomed to SMC at work were predicted to be better off when the crisis started. | We collected a nationally representative sample of Finnish workers before (N = 1308) and during (N = 1081) the COVID-19 crisis. Outcome measures included technostress and work exhaustion. Multilevel linear mixed-effects regression models investigated formal and informal SMC at work. Covariates included cyberbullying at work, social media usage, personality, occupational status and sociodemographic factors. | Results showed that formal SMC increased and predicted higher technostress. However, technostress and work exhaustion decreased among workers already accustomed to using SMC at work before the crisis. The results indicate a disparity in workers’ resilience during remote work and highlight a need for organizational level support. |
| 3 | (Kaltenegger et al., 2020) | 2020 | GERMANY | Association of working conditions including digital technology use and systemic inflammation among employees: Study protocol for a systematic review. | SCOPUS | The objective of this investigation will be to evaluate the association of working conditions including digital technology use and systemic inflammation among employees. | The authors designed and registered a study protocol for a systematic review of randomized controlled trials and prospective non-randomized studies (e.g., cohort, interrupted time series, or before-after studies). We will include studies conducted among adult workers reporting associations of working conditions and inflammatory activity. | The study methodological quality (or bias) is appraised using appropriate tools. The results are described qualitatively. Random effects meta-analysis was conducted, if feasible and appropriate. Additional analyses were performed to explore potential sources of heterogeneity. This systematic review and meta-analysis provide a synthesis of studies evaluating the association of working conditions and systemic inflammation. |
| 4 | (Brooks et al., 2020) | 2020 | USA, CHINA | Technology addictions and technostress: An examination of the US and China. | SCOPUS | In today's technology-centric world, people are becoming increasingly dependent on the Internet. The most common use of the Internet is through social media, which is used to communicate, share, collaborate, and connect. However, continued usage of a hedonic system can be linked with compulsion or addiction. Since problematic usage/behaviors can lead to negative outcomes, this study aims to determine differential effects of Internet and social media addictions on social media-related technostress. | This is examined in two different cultures: The U.S. and China. | The results support the association between the Internet and social media addictions with increases in social media-related technostress. Additionally, these effects are moderated by culture. Implications for research and practice are discussed along with future directions for this stream. |
| 5 | (Estrada-Muñoz et al., 2021) | 2021 | CHILE | Technostress of Chilean teachers in the context of the COVID-19 pandemic and teleworking. | SCOPUS | The lockdown imposed due to the COVID-19 pandemic prompted a rethink of the teaching-learning process, with teachers responding to the situation without prior planning using their own resources. This article shows the levels of technostress in primary and secondary education teachers in Chile, in the context of educational telework that Chile has adopted in connection with the health crisis by COVID-19. | The information has been collected with the use of the RED-TIC scale, previously used in this country, whose validity and reliability of the instrument has been treated, for this case, with confirmatory factorial analysis (CFA) with a national coverage sample of 3006 teachers. | The results show that 11% of teachers reveal techno anxiety and 7.2%, techno fatigue. Combining both manifestations, we find that 6.8% of teachers are techno stressed. Finally, fatigue and anxiety factors are higher for female teachers. |
| 6 | (Chou and Chou, 2021) | 2021 | TAIWAN | A multigroup analysis of factors underlying teachers’ technostress and their continuance intention toward online teaching | SCOPUS | The objective of this study was to analyze the factors underlying the intention to continue online teaching beyond the COVID-19 pandemic. | The person-environment fit theory was used to develop a survey for the study. An open-ended question appended to the survey helped to gather further thoughts from teachers on sustainable online teaching. | Teachers have experienced technostress in mandated online teaching during the pandemic. Privacy concerns aggravate teachers’ technostress in online teaching. Self-efficacy in online pedagogy would alleviate teachers’ technostress. School support matters less in teachers’ intentions to continue online teaching. Technostress plays a mediating role in bridging several relationships. The structural equation model reveals that teachers’ technostress is associated with their privacy concerns and self-efficacy in delivering effective instruction online. The multigroup analysis further demonstrates that technostress, self-efficacy and school support are related to teachers’ intentions to continue teaching online to different extents at different teaching levels. The responses to the open-ended question reveal that teachers’ preference for online teaching lies in adequate teaching resources and flexibility. |
| 7 | (Estrada Araos and Gallegos Ramos, 2022) | 2022 | CHILE | Tecnoestrés en el contexto educativo: Un problema emergente durante la pandemia COVID-19. | SCOPUS | This article reveals the levels of technostress among primary and secondary education teachers in Chile in the context of online teaching adopted by Chile in response to the COVID-19 public health crisis. | The sample consisted of 154 students of both sexes from the professional education, career and was determined by means of a stratified probabilistic sampling. The instrument used for data collection was the Scale of Attitudes towards virtual education, which has the required reliability and validity. | Based on the results, it was shown that the majority of students present an attitude of indifference towards virtual education and statistically significant differences were found between the sex and age of the students. |
| 8 | (Gabr et al., 2021) | 2021 | EGYPT | Effects of remote virtual work environment during COVID-19 pandemic on technostress among Menoufia University Staff, Egypt: A cross-sectional study. | SCOPUS | The objective of this study is to study technostress and challenges of remote virtual work environment among university staff members at Menoufia University, Egypt. A cross-sectional study was conducted over Menoufia University academic staff members in Egypt. The participants were chosen from both practical and theoretical colleges in Menoufia University using a multistage random sample. Tarfadar technostress questionnaire was used. Cortisol blood level was measured for all participants. | This study included 142 participants. The mean age of the group was 36.32±6.41 years. 52.1 percent worked in practical colleges, and 60.6% were lecturers or higher. Their mean cortisol level was 15.61±7.07mcg/dl. Participants who were females, reside in rural areas, held a lecturer or higher position, had poor work-environment WiFi, and lacked technical training had significantly higher levels of technostress subscales. | Most of the technostress subscales were significantly correlated with age and blood cortisol levels. The predictors of work overload in multivariate regression were female gender and a work environment with poor WiFi. Female gender, theoretical colleges, being lecturer or higher, and poor WiFi were the predictors for invasion. Among university staff members, technostress was found to be evident. High levels of technostress were significantly influenced by age, higher professions, female gender, and a bad workplace environment. |
| 9 | González-López et al., 2021) | 2021 | SPAIN | Overwhelmed by technostress? Sensitive archetypes and effects in times of forced digitalization. | SCOPUS | This paper explores technostress and its dimensions, assessing the relationship with possible negative effects in the individual, social and professional spheres. The analysis, based on an exploration of a system of archetypes of social media use, presents insights into contemporary technostress management as a new approach offering opportunities to optimize prevention plans. Pearson’s correlation coefficients and structural equation modeling based on partial least squares (SEM-PLS) were the methods used to fulfil the study objectives. | The study uses self-reporting by undergraduate students in Spain (n = 337), who were forced to lead their academic lives entirely through technology due to social distancing, a public health measure adopted to reduce the spread of COVID-19. | The results provide valid, reliable measures indicating the major impact of technostress on students’ individual spheres and show a significant relationship between user type and techno-anxiety. |
| 10 | (Becker et al., 2022) | 2022 | GERMANY | Mitigating the negative consequences of ICT use: the moderating effect of active-functional and dysfunctional coping | SCOPUS, WoS | This study aims to measure the moderating effect of active-functional and dysfunctional coping in relation to the negative consequences of ICT use. | The moderating effects of two different reactive coping strategies – active-functional and dysfunctional – were investigated in a sample of 3,362 German knowledge workers. | A competitive mediation effect was found, where the direct effect of demands on productivity is in the opposite direction to the indirect effect. Both active-functional and dysfunctional coping reduce the extent to which demands lead to strain. The contribution of this paper to technostress research is discussed and implications for future research are suggested. Recommendations for employers and employees are highlighted. |
| 11 | (Sinha et al., 2021) | 2021 | INDIA | Technology: Saving and enriching life during COVID-19 | WoS, SCOPUS | The study was primarily focused on three objectives. Firstly, it attempted to understand the ways in which senior citizens made use of social networking sites and various digital platforms to manage their lives better. Secondly, it analyzed the technology adoption process, and finally, it examined the extent of the impact of technology on elderly people’s lives and the permanent nature of this change. | The review is based on online data on the rise in the proportion of elderly people (age) using these platforms (social networks and ICTs for communication) and on recent studies analyzing the effects of the COVID-19 pandemic on senior citizens.  Suitable participants were identified via convenience sampling and contacted by telephone. Subsequently, the scripts were analyzed in search of the key questions raised in the study. | The analysis clearly points to an increase in digital life among elderly people. The technology adoption process progressed in stages, from complete confusion to relative ease, significantly reducing elderly people’s loneliness and bringing about a relatively stable change to their way of life. |
| 12 | (Carillo et al., 2021) | 2021 | FRANCE | Adjusting to epidemic-induced telework: Empirical insights from teleworkers in France. | WoS | The COVID-19 pandemic has given rise to unprecedented challenges, with far-reaching implications for the ways in which people live and work. | This paper develops an epidemic-induced telework adjustment model derived from the theory of work adjustment and the interactional model of individual adjustment. It is tested on a sample of 1,574 teleworkers in France. | The results point to a significant influence of crisis-specific variables such as professional isolation, telework environment, increased workload and stress. |
| 13 | (Spagnoli et al., 2020) | 2020 | ITALY | Workaholism and technostress during the COVID-19 emergency: the crucial role of leaders on remote working | WoS | The present study aimed to explore the role of authoritarian leadership in a sample of 339 administrative university employees who worked either completely from home or from home and the workplace. | The study examined the moderating effect of a manager on this relationship and the connections between workaholism and technostress through conditional process analysis. | Results pointed out that high authoritarian leadership had an enhancing effect, whereas low authoritarian leadership had a protective effect on the relationship between workaholism and technostress, only in the group of complete remote workers. Thus, authoritarian leadership should be avoided and training leaders to be aware of its effect appears to be essential. Limitations, future directions for the study, and practical implications are also discussed. |
| 14 | (Zito et al., 2021) | 2021 | ITALY | Does the end justify the means? The role of organizational communication among work-from-home employees during the COVID-19 pandemic. | WoS | This study aimed to detect, in such a period of crisis and changes, the role of organizational communication considering the mediating role of both technostress and self-efficacy, with psycho-physical disorders as outcome. | The research involved 530 workers working from home. A Structural Equations Model was estimated, revealing that organizational communication is positively associated with self-efficacy and negatively with technostress and psycho-physical disorders. | As mediators, technostress is positively associated with psycho-physical disorders, whereas self-efficacy is negatively associated. As regards mediated effects, results showed negative associations between organizational communication and psycho-physical disorders through both technostress and self-efficacy. This study highlighted the potential protective role of organizational communication that could buffer the effect of technostress and enhance a personal resource, self-efficacy, which is functional to the reduction of psycho-physical disorders |
| 15 | (Kim and Kang, 2020) | 2020 | KOREA, REPUBLIC OF | The impact of technostress on counter-productivity | WoS | COVID-19 is quickly spreading around the world and accelerating a shift towards home-based teleworking environments. Many companies, however, are afraid of reduced productivity due to insufficient readiness to work in new technological environments. Therefore, identifying the factors that can improve productivity in home-based teleworking environments is a very important research topic. | This study investigated the effect of technostress on counter-productivity. Unlike the previous studies, this study was made with the survey for firm employees of Daegu region, and both counter-productive work behavior and innovation resistance were used in this study. | The empirical result means that technostress positively affects both counter-productive work behavior and innovation resistance. Because technostress increases both counter-productive work behavior and innovation resistance, systematic management for firm employees is needed in time adopting information and communication technologies. |
| 16 | (Hwang et al., 2022) | 2022 | KOREA, REPUBLIC OF | Impact of regulatory focus on security technostress and organizational outcomes: The moderating effect of security technostress inhibitors. | WoS, SCOPUS | The study examined the moderating effect of a manager on this relationship and the connections between workaholism and technostress using conditional process analysis. | A survey questionnaire was developed, collecting 346 responses from various organizations, which were analyzed using the structural equation model approach with AMOS 22.0 to test the proposed hypotheses. | The results indicate support for both the direct and moderating effects of security technostress inhibitors. Moreover, a negative relationship exists between promotion-focused employees and facilitators of security technostress, which negatively affects strains (organizational commitment and compliance intention). |
| 17 | (Panisoara et al., 2020) | 2020 | ROMANIA | Motivation and continuance intention towards online instruction among teachers during the COVID-19 pandemic: the mediating effect of burnout and technostress. | WoS | Drawing on the Self-Determination Theory (SDT), Job Demands-Resources Model (JD-R), and Technology Acceptance Model (TAM), the research model used in this study includes technological pedagogical knowledge (TPK) self-efficacy (SE), intrinsic (IM) and extrinsic (EM) work motivation, and occupational stress (OS) (i.e. burnout and technostress, which were examined in tandem) as key dimensions to explain intentions among in-service teachers to continue to use online-only teaching (CI). | Data for the research model were collected from 980 in-service teachers during the COVID-19 outbreak between April and May 2020. Overall, the structural model explained 70% of the variance in teachers’ CI. Motivational practices were directly and indirectly linked with CI through OS. | The findings showed that IM has the most direct significant effect on teachers’ CI, followed by TPK-SE and OS as significant but less relevant predictors. IM was positively associated with TPK-SE and negatively associated with EM. The results offered valuable insight into how motivation constructs were related to OS and to an improved understanding of online teaching in an unstable work context, in order to support teachers in coping with remote work. |
| 18 | (Christ-Brendemuhl and Schaarschmidt, 2020) | 2020 | KOREA, REPUBLIC OF | The impact of service employees’ technostress on customer satisfaction and delight: A dyadic analysis. | WoS | This study aims at investigating to which extent technological demands arouse FLE technostress and how the latter affects customer satisfaction and delight with the FLE as well as electronic word-of-mouth. With conservation of resources (COR) as a theoretical framework, the authors use the job demands-resources (JD-R) model to examine the influence of technology on service interactions. | Dyadic data gathered from a field study in 73 full-service restaurants that use frontline service technology, span 147 FLEs, and 373 corresponding customers. | The results confirm that technology-induced job demands lead to FLE technostress, while optimism towards technology reduces the latter and buffers the effect of role ambiguity on technostress. Technostress reduces both customer satisfaction and delight with the FLE. The findings emphasize the challenge of effectively managing technology-induced job demands in organizational frontlines. |
| 19 | (Califf and Brooks, 2020) | 2020 | USA | An empirical study of techno-stressors, literacy facilitation, burnout, and turnover intention as experienced by K-12 teachers. | WoS | This paper builds and empirically tests a model of technostress in the context of kindergarten through 12th grade (K-12) education. Technostress is framed conceptually as a process that includes techno-stressors, strain, and outcomes, as well as mechanisms that can reduce the techno-stressors and strain variables. | The model is tested empirically using survey data from 416 K-12 teachers employed in the United States. | The effects of five techno-stressors—techno-complexity, techno-insecurity, techno-invasion, techno-overload, and techno-uncertainty—on strain in the form of burnout, and the impact of burnout on turnover intention, are investigated. The mitigating effect of literacy facilitation on the five techno-stressors and burnout is also tested. Results indicate that techno-insecurity, techno-invasion, and techno-overload significantly increase burnout, and literacy facilitation can mitigate the negative impact of techno-complexity, techno-insecurity, techno-invasion, techno-overload, and burnout. Burnout also has a significant positive effect on turnover intention. |
| 20 | (Califf et al., 2020) | 2020 | USA | The bright and dark sides of technostress: A mixed-methods study involving healthcare IT. | WoS | Technostress is often characterized by researchers as negative, or as being on the “dark side” of technology. However, a broader reading of the stress literature suggests that technostress may be both positive and negative and can therefore have a “bright side” in addition to a dark side. The objective of this study is to conceptualize a holistic technostress process that includes positive and negative components of technostress embedded in two subprocesses: the techno-eustress subprocess and the techno-distress subprocess, respectively. | The study instantiates this holistic technostress model through a sequential mixed-methods research design in the context of HIT. Phase 1 of the design is a qualitative, interpretive case study involving interviews with 32 nurses. | Based on the findings from the case study, the paper builds a research model that operationalizes the concepts embedded in the holistic technostress model and identifies contextually relevant challenge and hindrance techno stressors and outcomes. In Phase 2, the research model is empirically validated by analyzing survey data collected from 402 nurses employed in the United States. |

**Source:** Compiled by the authors **(2022)**
